# Apis

**Published:** 1888-08

**Authors:** W. J. Hunter Emory


### APIS.

This remedy will probably be found most frequently indicated in the convulsions of children, dependent upon some form of cephalic disturbance, and in acute apoplexias.

Characteristics.—Soporous conditions, interrupted by occasional frequent sudden startings, accompanied by a *shrill cry, almost amounting to a shriek.* Extreme restlessness at night. General aggravation toward morning. *Urine suppressed* or *scanty, red,* and *hot.*

Child *awakens from a sleep* with a *sudden, shrill, piercing cry,* and convulsions immediately ensue. Irritable, irascible mood; more rarely dejection.

All troubles arise and centre in jealousy.

Child lies in torpor; delirious, sudden shrilling cries, squinting, grinding the teeth, boring head in pillow; one side twitching, the other paralyzed; head wet from sweating; urine scanty, red, hot, or milky; big toe turns up; nausea, breath offensive; tongue red, sore; hydrocephaloid; eyes red, head hot, shrill shrieks, tongue dry, skin dry, hands cold and blue; urine suppressed, abdomen tender; mucous, purulent, offensive, involuntary diarrhœa; head large and eyes prominent; fontanelles reopen at from six to ten months, with great restlessness, and frequent putting of the hand to head.

Bores head into pillow.

Bending back and rolling of the head. Œdematous swellings in general, particularly about face and eyes. Photophobia, pupil drawn to one side in an oblong direction; double vision.

Quivering and twitching of eyelids and eyeballs, particularly the left. Anxious expression of face, or an expression of terror or apathy, which may be pale and œdematous, or flushed.

Spasmodic snapping of lower jaw; generally absence of thirst; great debility.

Single jerks of one or more limbs frequently repeated.

W. J. HUNTER EMORY.

